# Unveiling new perspective of phylogeography, genetic diversity, and population dynamics of Southeast Asian and Pacific chickens

**DOI:** 10.1038/s41598-022-18904-3

**Published:** 2022-08-26

**Authors:** Cyrill John P. Godinez, John King N. Layos, Yoshio Yamamoto, Tetsuo Kunieda, Monchai Duangjinda, Lawrence M. Liao, Xun-He Huang, Masahide Nishibori

**Affiliations:** 1 Laboratory of Animal Genetics, Graduate School of Integrated Sciences for Life, Hiroshima University, Higashi-Hiroshima, 739-8528 Japan; 2 Department of Animal Science, College of Agriculture and Food Science, Visayas State University, Visca, Baybay City, Leyte 6521 Philippines; 3 College of Agriculture and Forestry, Capiz State University, Burias, Mambusao, Capiz, 5807 Philippines; 4 Faculty of Veterinary Medicine, Okayama University of Science, Imabari, Ehime 794-8555 Japan; 5 Department of Animal Science, Faculty of Agriculture, Khon Kaen University, Khon Kaen, 40002 Thailand; 6 Laboratory of Aquatic Botany, Graduate School of Integrated Sciences for Life, Hiroshima University, Higashi-Hiroshima, 739-8528 Japan; 7 School of Life Sciences, Jiaying University, Meizhou, 514015 China

**Keywords:** Evolution, Genetics

## Abstract

The complex geographic and temporal origins of chicken domestication have attracted wide interest in molecular phylogeny and phylogeographic studies as they continue to be debated up to this day. In particular, the population dynamics and lineage-specific divergence time estimates of chickens in Southeast Asia (SEA) and the Pacific region are not well studied. Here, we analyzed 519 complete mitochondrial DNA control region sequences and identified 133 haplotypes with 70 variable sites. We documented 82.7% geographically unique haplotypes distributed across major haplogroups except for haplogroup C, suggesting high polymorphism among studied individuals. Mainland SEA (MSEA) chickens have higher overall genetic diversity than island SEA (ISEA) chickens. Phylogenetic trees and median-joining network revealed evidence of a new divergent matrilineage (i.e., haplogroup V) as a sister-clade of haplogroup C. The maximum clade credibility tree estimated the earlier coalescence age of ancestral D-lineage (i.e., sub-haplogroup D2) of continental chickens (3.7 kya; 95% HPD 1985–4835 years) while island populations diverged later at 2.1 kya (95% HPD 1467–2815 years). This evidence of earlier coalescence age of haplogroup D ancestral matriline exemplified dispersal patterns to the ISEA, and thereafter the island clade diversified as a distinct group.

## Introduction

The domestication of animals has led to important shifts in human demographics that helped shape early human societies. Domestic chicken (*Gallus gallus domesticus*) is one of the world’s most widely distributed domestic animal species. It plays a key role in human societies as the largest source of animal protein^1,2^ and as a significant factor in socio-cultural development^3^. Since domestication, chickens have been distributed throughout various countries and continents, resulting in a wide range of chicken breeds today^4,5^. Despite their global distribution, studies on the chicken domestication process and translocation history remain obscure. Modern biological and zooarchaeological approaches suggest that chicken domestication probably occurred across southwest China and Southeast Asia, involving one or more wild progenitors across their native geographical range^6–12^. Subsequently, domestic chickens have been translocated out of their domestication centers to every inhabited region by human migration and trade expansion. This led to the evolution of subpopulations of chickens in response to natural selection pressure and selective breeding for adaptation to the variety of agro-ecological conditions^13^.

Southeast Asia (SEA), being the most geographically complex tropical region on Earth, has given rise to a diverse and highly endemic avifauna^14,15^. The emergence of agricultural societies harboring domesticated animals allowed a remarkable expansion of genetically divergent domesticated populations, a case seen in chickens that likely followed a commensal route of the domestication process^16^. In addition, the favorable seasonal weather patterns and vegetation in the region made it a suitable environment for chicken dispersal^10,17,18^. Recently, several DNA sources and molecular strategies were used to resolve chicken phylogeny and their genetic expansion from their wild progenitors^11,19–23^. However, major challenges from the zooarchaeological perspective remain as there are only a few reports of chicken remains in SEA^24^, and prehistoric exploitation has yet to be elucidated^25^. Such evolutionary links would likely provide a better understanding of the evolutionary history and population dynamics of the world’s most common farm animal.

Early studies reconstructing the matrilineal history of domestic chickens based on mitochondrial DNA (mtDNA) analysis supported a monophyletic origin of the red junglefowl (RJF), which serves as the primary wild ancestor of domestic chickens^26,27^. However, from the early twenty-first century, numerous mtDNA analyses suggested multiple domestication events^7,8,28^ and the possibility of different *Gallus* species contributing to the genetic makeup of domestic chickens^20,22,29,30^. Moreover, recent genome-wide data linked domestic chickens most closely to the Southeast Asian subspecies *G. g. spadiceus*, which locally interbred with other subspecies across South and Southeast Asia^11^. Mitochondrial DNA D-loop variation has been extensively used to gain a better understanding of chicken populations, types, evolutionary relationships, and domestication history. Chickens have been classified into eight highly divergent maternal haplogroups (A–G, V) and six rare haplogroups (H–I, W–Z)^8,31^. Major haplogroups A and B were ubiquitously distributed in Asian regions, whereas haplogroup E was widely distributed in Europe, the Middle East, Africa, and South America^8,19,32,33^. Haplogroup C was spread over East Asia, whereas haplogroup F was restricted to Yunnan, China and Myanmar^8,31,34^. Haplogroup D was mostly found in SEA and Pacific populations^8,35–37^. The knowledge of population studies on the genetic diversity, population structures, and demography is essential to understanding the role of past and present evolutionary processes of chickens over the course of domestication.

Here, we generated complete mtDNA D-loop sequences of chickens from mainland SEA (Cambodia, Laos, Thailand, and Myanmar), the Philippines, and Fiji, spanning a geographical transect that is believed to encompass possible translocations of this taxon in the region. By combining these newly generated sequence data with previously published data from ISEA chickens (the Philippines and Indonesia) and Pacific chickens, as well as other neighboring chicken populations in Asia, we sought to obtain an updated perspective of the matrilineal phylogeny and demographic events that shaped the genetic diversity of SEA and Pacific chickens. Specifically, we would like to estimate their lineage-specific divergence, genetic similarities, and differentiation within and between continental and island populations.

## Results

### Haplotype variation and genetic diversity

We analyzed complete mtDNA control region sequences of chickens from Cambodia (*n* = 173), Laos (*n* = 63), Thailand (*n* = 25), Myanmar (*n* = 78), the Philippines (*n* = 6), and Fiji (*n* = 24) generated in this study and including previously published sequences from the Philippines (*n* = 129), Indonesia (*n* = 10), and Pacific (*n* = 11). A total of 133 haplotypes were identified, with 70 variable sites consisting of 10 singletons and 60 parsimony informative sites. We documented 82.7% geographically unique haplotypes, while 17.3% of haplotypes were shared transregionally across SEA, suggesting high polymorphism among the studied individuals. Island populations (i.e., Philippine and Pacific chickens) accounted for 28% of all unique haplotypes identified, while 72% were unique to continental populations. Summary of observed polymorphic sites and haplotype variations are presented in Supplementary Tables S1–S2.

The indices of genetic diversity for each geographic population are shown in Table 1. Undoubtedly, haplotypic and nucleotide diversity was very high in SEA chicken populations. The mainland Southeast Asia (MSEA) chickens had higher total haplotypic diversity (*Hd* = 0.963 ± 0.005) and nucleotide diversity (*π* = 0.00782 ± 0.00398) than the Island Southeast Asia (ISEA) chickens (*Hd* = 0.942 ± 0.009; *π* = 0.00466 ± 0.00249), although no major differences were observed. The highest value of *Hd* and *π* was found in Thai chickens (with 72% RJF population in our dataset), whereas the least was observed in Pacific chickens. These results should be taken with caution, given the Pacific chickens have a relatively small sample size, and the Thai chicken dataset was predominated with RJFs (18/25) (Supplementary Tables S1–S2). Thus, the genetic diversity is usually higher than that of the domestic chicken populations. Remarkably, the Thai chickens had a high number of haplotypes (Ht = 19) in 25 individuals examined, suggesting a diverse population in the region. Similarly, intraclade diversity indices indicated high haplotype and nucleotide diversity of haplogroup D than all other major haplogroups classified in SEA and the Pacific chickens (Supplementary Table S3).

**Table 1:** Genetic diversity indices of Southeast Asian and Pacific chicken populations estimated using complete mtDNA D-loop sequences.

Region	Molecular diversity indices				Neutrality tests	
	N	Ht	*Hd*	*π*	Tajima’s *D*	Fu’s *F*_*S*_
Cambodia	173	54	0.889 ± 0.019	0.00585 ± 0.00030	− 0.3677	− 22.826**
Laos	63	27	0.935 ± 0.013	0.00678 ± 0.00028	0.7429	− 4.4319
Thailand	25	19	0.953 ± 0.029	0.00912 ± 0.00051	0.5534	− 4.0472*
Myanmar	78	35	0.922 ± 0.016	0.00837 ± 0.00020	0.7659	− 6.6700
Philippines	135	36	0.900 ± 0.013	0.00435 ± 0.00027	0.0318	− 11.1450**
Fiji	24	7	0.725 ± 0.077	0.00380 ± 0.00036	1.1958	2.1475
Pacific *overall*^a^	35	15	0.862 ± 0.047	0.00386 ± 0.00215	− 0.5632	− 2.8581
MSEA *overall*^b^	339	91	0.963 ± 0.005	0.00782 ± 0.00398	− 0.2515*	− 24.037**
ISEA *overall*^c^	145	41	0.942 ± 0.009	0.00466 ± 0.00249	− 0.2570	− 16.235**

^a^Included published sequences of Pacific chickens (*n* = 11).

^b^Combined sequences representing MSEA (Cambodia, Laos, Thailand, and Myanmar chickens).

^c^Combined sequences representing ISEA (Philippine chickens and database sequences of Indonesian chickens; *n* = 10).

*N* number of sequences, *Ht* number of haplotypes, *Hd* haplotype (gene) diversity, *π* nucleotide diversity.

* *p < 0.05*; ** *p < 0.01*.

### Phylogeography and genetic affinities of continental and island SEA chickens

The sequences generated in the present study and the reference sequences that represent chicken mtDNA control region-based haplogroup nomenclatures were used to reconstruct the matrilineal phylogeny (Supplementary Tables S1, S4). Pioneering molecular phylogenetic studies based on mtDNA control region and mitogenomes revealed fourteen haplogroups (A-I and V-Z) of chicken worldwide^7,8,19,31^. Divergent haplogroups D and V showed enigmatic phylogeny resolution and previously claimed to have been distributed in ISEA and Thailand, respectively^7,31,36,37^.

**Figure 1:**
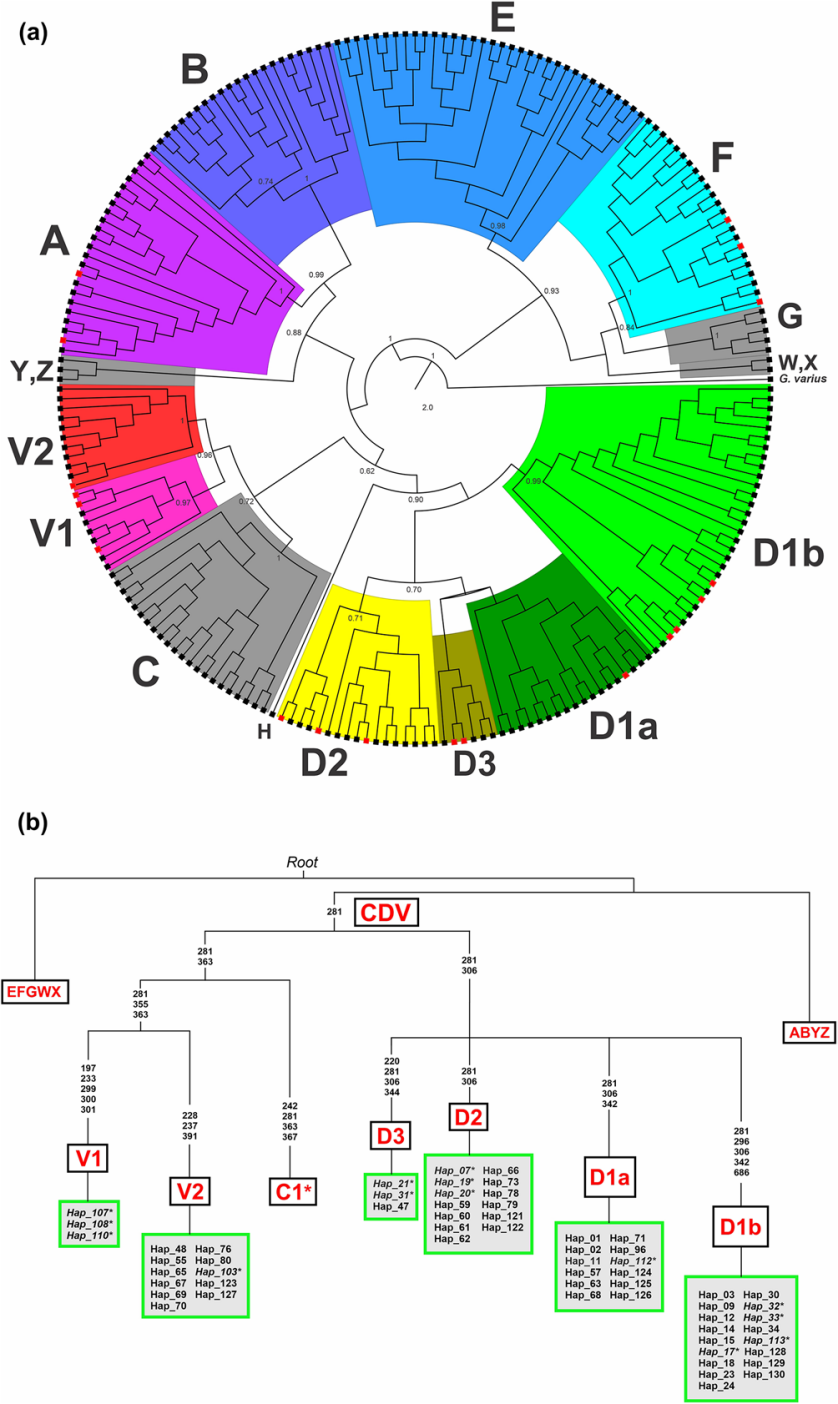
(**a**) Bayesian phylogenetic tree of complete mtDNA D-loop nucleotide sequences of Southeast Asian and Pacific chickens. The tree was constructed together with database sequences defined by Huang et al.^31^ (Supplementary Table S4). Node labels correspond to posterior probability support values. Identified haplogroups are assigned with color codes, while grey color is assigned for reference haplogroup nomenclatures with no classified samples. Tips highlighted in red indicate red junglefowl. (**b**) Schematic classification tree showed reclassified macrohaplogroup CDV. The nucleotide positions were scored relative to the reference sequence NC_040970. Mutational motifs (transitions) are shown on the branches. Haplotypes in italics and asterisk indicate red junglefowl. Tree file was visualized and edited in FigTree v1.4.4. (http://tree.bio.ed.ac.uk/software/figtree/).

In this study, model-based maximum likelihood and Bayesian phylogenetic analyses produced concordant topologies and comparable branch lengths of the tree (Fig. 1a; Supplementary Figs. S2–S3). Major clades have strong SH-aLRT and UFBoot supports for the ML tree and significant posterior probability support for the Bayesian tree. Minor differences involved only some rearrangements of terminals for haplotypes: Hap_60, Hap_61, Hap_62, Hap_66, and Hap_122, as they clustered with haplogroup D1 (i.e., sub-haplogroup D1a) in the ML tree (Supplementary Fig. S2), while grouped with haplogroup D2 in the Bayesian tree (Supplementary Fig. S3).

Phylogenetic analyses grouped the MSEA chickens into major haplogroups A, B, D, E, and F, with the evidence of newfound haplogroup V as a sister-clade of haplogroup C (Fig. 1a; Supplementary Fig. S2). Haplogroup V, classified by ancestral mutation motifs A281G, T355C, and C363T, was further subdivided into two sub-haplogroups (Fig. 1b). Here, we documented evidence of sub-haplogroup V2 (classified by unique mutational motifs: C228T, A237G, C391T) only identified in Cambodian and Laotian domestic chickens, and one haplotype of Thai RJFs (*G. g. gallus*) (Hap_103) at the basal position of the sub-clade (Supplementary Fig. S3; Supplementary Table S1). Evidence of sub-haplogroup V1 observed predominantly in Thai RJFs (Hap_107, Hap_108, and Hap_110) shared commonality to the reclassified haplogroup V of RJFs in Thailand and Cambodia^31^ (Supplementary Fig. S3). Interestingly, both model-based phylogenetic trees revealed ancestral lineage of haplogroup D2 from MSEA chickens, mostly observed in Cambodian chickens (38.7%) and some low frequency of Laotian (7.9%) and Thai chickens (8.0%), while remaining undetected in Myanmar chickens (Fig. 1a; Supplementary Fig. S2; Supplementary Table S1). Haplogroups A and B have wide geographical distribution all over SEA, while haplogroup F was prevalent among Myanmar chickens (34.6%), with some low frequency detected in Thai chickens (12.0%).

Likewise, ISEA chickens (i.e., the Philippines and Pacific) have a shared genetic affinity for predominant haplogroup D1. Godinez et al.^37^ previously characterized this island sub-group as the “Philippine-Pacific sub-clade.” This sub-clade is defined by five unique mutational motifs, A281G, C296T, T306C, A342G, and G686A, and includes diagnostic motifs from the downstream region of the complete mtDNA control region sequence (Fig. 1b; Supplementary Table S1). These findings also correspond to the diagnostic motifs (SNPs: A281G, C296T, T306C, A342G) of Polynesian chicken ancient DNA^36^.

**Figure 2:**
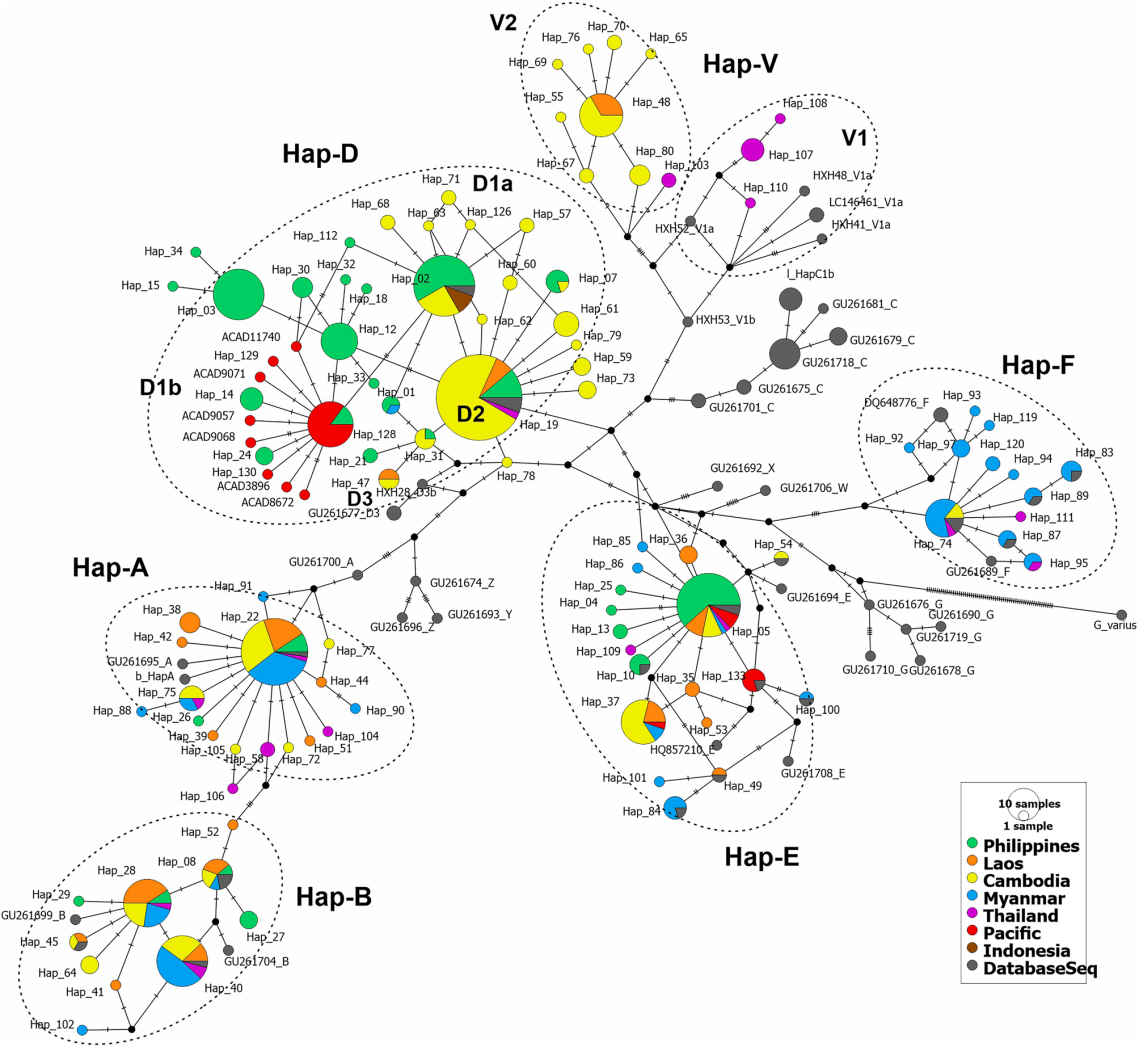
Median-joining network of the complete mtDNA D-loop region (1232 bp) depicting the evolutionary relationship of MSEA and ISEA chicken populations. The area of each circle is proportional to the frequency of the corresponding haplotypes. The length of the branch connecting to other haplotypes corresponds to mutational positions. The figure was created using PopArt v1.7 software (http://popart.otago.ac.nz/).

Consistent classification of the major mitochondrial lineages of SEA chickens was also depicted in the median-joining (MJ) network analysis (Fig. 2). Notably, haplogroup V lineage was separated from haplogroup D and haplogroup C with nine and seven mutational sites, respectively. Within the haplogroup V lineage, newly classified sub-haplogroup V2 was separated from sub-haplogroup V1 with four mutational signatures. The geographical-specific MJ network analyses exhibited a close transregional evolutionary relationship of MSEA chickens in major haplogroups except for haplogroup F, which was predominated in Myanmar chickens (Supplementary Fig. S4a–d). Similarly, the Philippine and Pacific chickens also shared closely related haplotypes classified under sub-haplogroup D1b (Supplementary Fig. S4e–f).

### Population structure and genetic differentiation

**Figure 3:**
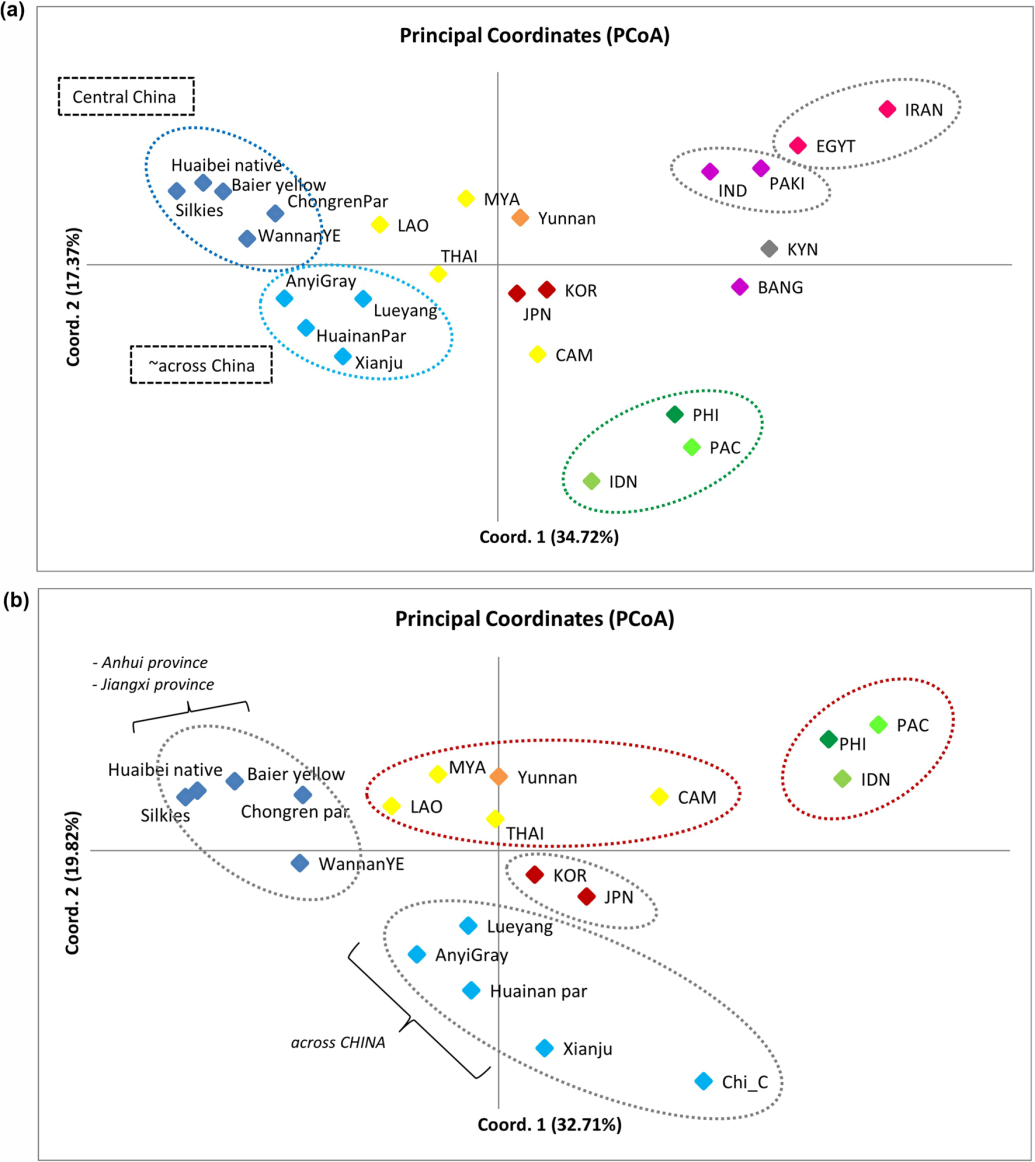
(**a**) PCoA plot of population pairwise *F*_*ST*_ values of SEA and Pacific chickens, together with other chicken populations from East Asia (i.e., China, Japan, and Korea), South Asia (i.e., Bangladesh, Pakistan, and India), Africa (i.e., Egypt and Kenya), and Middle East (Iran). The geographic origins of populations are shown by different colors (yellow: MSEA, green: ISEA and Pacific, blue: China, red: Japan and Korea, purple: South Asia, pink: Egypt and Iran, and gray: Kenya). (**b**) PCoA plot of population pairwise *F*_*ST*_ values of SEA and Pacific chickens after removing South Asian and Middle Eastern chicken sequences.

We carried out a multivariate approach to complement the phylogenetic analyses to assess further the relationships among and between geographical populations, including database sequences of East Asia, South Asia, and Middle East chickens (Supplementary Table S5). The result of the PCoA distinguished population substructure between mainland and island SEA chickens along the first two axes, which accounted for 52.09 or 52.53% variation (Fig. 3a–b). A homogenous subgroup was observed within island populations, particularly among the Philippine and Pacific chickens (*F*_*ST*_ = 0.06936), while MSEA populations showed more a diverse assemblage, consistent with the phylogenetic analyses and haplogroup variations. In addition, we documented closer relationships between Myanmar chickens and Yunnan chickens than any other Chinese chicken population. The pairwise *F*_*ST*_ value confirmed that Myanmar and Yunnan chickens were not differentiated from each other (*F*_*ST*_ = 0.00816; *p value* < 0.01). Meanwhile, within MSEA chickens, transregional population substructures were observed ranging from 0.06895 between Laos and Thailand to 0.19202 between Cambodia and Myanmar (Supplementary Table S6). Interestingly, Cambodian chickens were situated halfway between other continental populations and ISEA chickens, supporting the basal affiliations of identified ancestral matriline (i.e., sub-haplogroup D2) depicted in both ML and BI phylogenetic trees. The PCoA plot also indicates a significant genetic differentiation and substructure between East Asian chickens and South Asian-Middle Eastern chickens, ranging from 0.14938 to 0.77115 (Fig. 3a; Supplementary Table S6). Similarly, we observed a close genetic affinity of Japanese and Korean chickens to the Chinese and MSEA chicken populations after removing South Asian and Middle Eastern chickens from the dataset (Fig. 3b).

Hierarchical AMOVA revealed that the majority of the variations (i.e., 79.21% between ISEA and MSEA chickens and 79.74% between MSEA and EA chickens) could be attributed to within-population differentiation, specifically chickens distributed across Southeast and East Asia (Table 2). Higher within-population variation was also observed within ISEA and Cambodian chickens. Likewise, no significant population genetic differentiation was found among groups of the island and mainland SEA chickens and groups of MSEA and East Asian chickens. These observed patterns of genetic differentiation from the partitioned variances among hierarchical groups reflect consistency established in the previous phylogenetic and PCoA analyses.

**Table 2:** Population genetic structure estimated from the AMOVA based on complete mtDNA D-loop sequences from (1) Philippine chickens, (2) Pacific chickens, (3) Indonesian chickens, (4) Cambodian chickens, (5) Laotian chickens, (6) Thailand chickens, (7) Myanmar chickens, and database sequences from East Asia (EA), South Asia (SA), and Middle East (ME).

Group	*N*	No. of popul-ation	No. of groups	Source of variation (%)		
				Among groups	Among populations within group	Within populations
No grouping	526	7	1	–	17.70*	82.30
A (ISEA-1,2,3 vs. MSEA-4,5,6,7)	526	7	2	9.06	11.73**	79.21**
a.1 (1,2,3 vs. 4)	360	4	2	5.92	7.38**	86.70**
a.2 (1,2,3 vs. 5)	250	4	2	24.37	6.06**	69.57**
a.3 (1,2,3 vs. 6)	212	4	2	22.59	6.32**	71.09**
a.4 (1,2,3 vs. 7)	265	4	2	26.12	5.20**	68.68**
B (ISEA vs. EA)	677	15	2	15.66*	18.63**	65.71**
C (ISEA vs. SA)	259	6	2	18.53*	8.14**	73.33**
D (ISEA vs. ME)	244	5	2	34.25*	5.63**	60.12**
E (MSEA vs. EA^a^)	829	16	2	1.17	19.09**	79.74**
F (MSEA vs. SA^a^)	411	7	2	10.86*	11.39**	77.75**
G (MSEA vs. ME^a^)	396	6	2	19.93*	10.57**	69.51**

^a^Database sequences retrieved from GenBank.

Significant fixation indices at * *p* < *0.05*; ** *p* < *0.01*.

### Demographic history and divergence time estimate

The simulations for neutrality tests indicated both MSEA and ISEA chickens deviated from neutrality (Table 1), which supported a demographic expansion. The negative and significant Tajima’s *D* and Fu’s *Fs* statistical values of MSEA chickens and significantly negative Fu’s *Fs* value of ISEA chickens provided evidence for population growth signatures in the Asia–Pacific region. To obtain a better inference of the demographic history of MSEA and ISEA chickens, we evaluated the changes in maternal effective population sizes (*N*_e_) at the different points along with the genealogical timescale. The Bayesian Skyline Plot (BSP) showed evidence of MSEA chicken populations experiencing an episode of population stasis during the early Holocene period, but *N*_e_ started to increase around 4000 years BP, and imminent population growth commenced about 3000–3500 years BP (Supplementary Fig. 5a–c). On the other hand, the Philippine and Pacific chickens later started to increase their *N*_e_ around 2500 years BP and 1500 years BP, respectively (Supplementary Fig. 5d–e). Looking into the individual geographical population, BSP indicated earlier population growth of Myanmar chickens (~ 4.0 kya) than the Cambodian and Laotian chickens (~ 3.0 kya). We did not run for demic demographic inference for the Thai chicken population because it violates the sample size parameter. Similarly, among within-island populations, Philippine chickens were observed to show increased *N*_e_ around 2.5 kya, while Pacific chickens have a much recent population growth expansion estimated at 1.0–1.5 kya (Supplementary Fig. S5).

**Figure 4:**
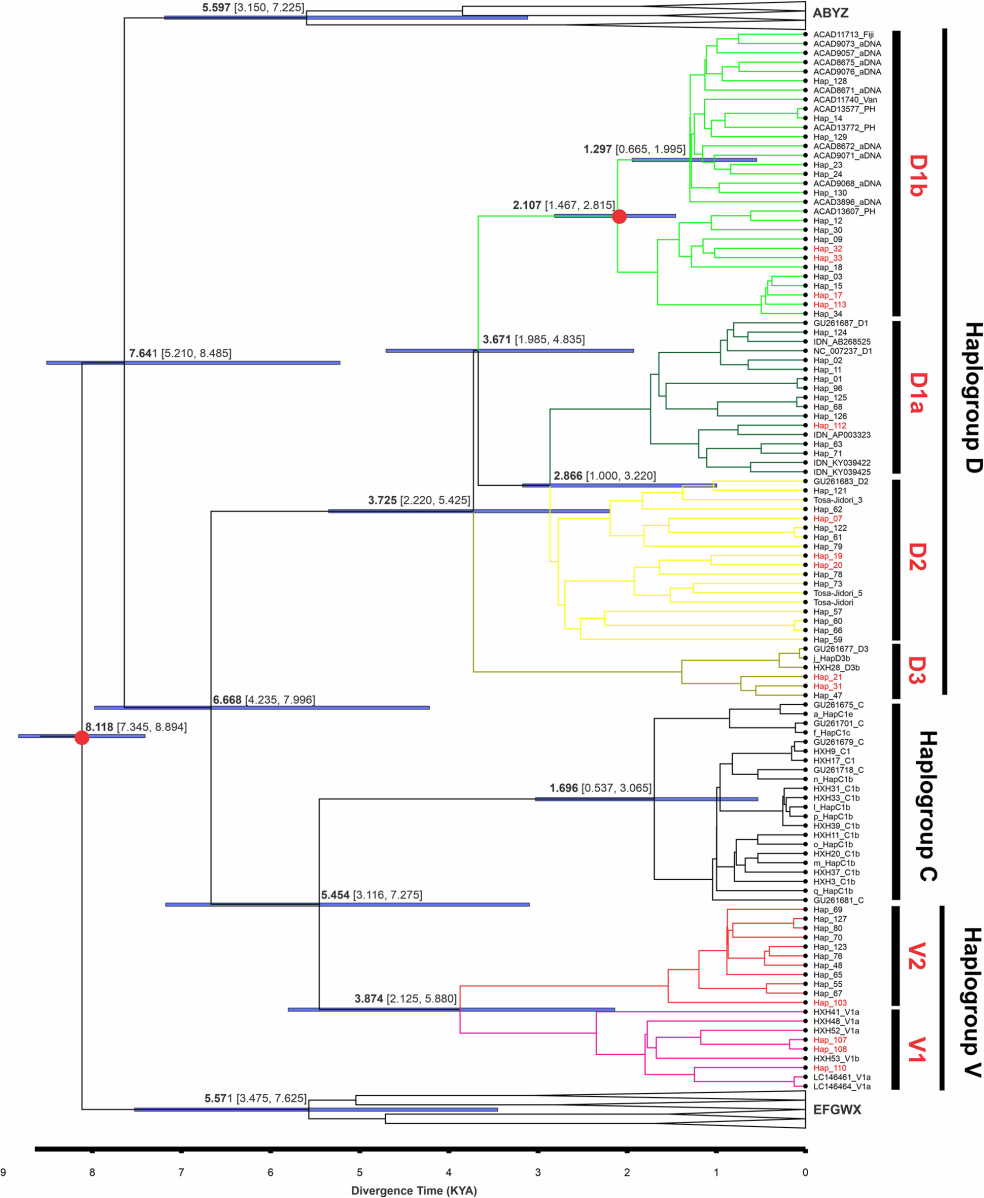
Time tree phylogeny depicting divergence time estimate based on primary and secondary calibration using BEAST2 v2.6.6. Red dots indicate the nodes with calibrations. Node labels indicate the median estimated divergence time, blue bars indicate the 95% HPDs. Tip labels highlighted in red indicate red junglefowl. Tree file was visualized and edited in FigTree v1.4.4. (http://tree.bio.ed.ac.uk/software/figtree/).

The maximum clade credibility (MCC) tree estimating the divergence time using a calibration method under an uncorrelated lognormal relaxed clock model revealed age estimates for biogeographically important nodes of haplogroups D and V in our dataset (Fig. 4c). The node age of macrohaplogroup CDV was estimated to be 6.67 kya with credibility intervals of 4235–7996 years (95% HPD). The coalescence age of sub-haplogroup D1b (PP = 1) was dated back to 2.1 kya (95% HPD 1467–2815 years) while diverging from the ancestral D-lineage approximately 3.7 kya (95% HPD 1985–4835 years). Haplogroup CV (PP = 0.80) diverged much earlier from macrohaplogroup CDV and coalesced around 5.5 kya (95% HPD 3116–7275 years) while succeeding divergence of haplogroup V (PP = 0.96) occurred around 3.9 kya (95% HPD 2125–5880 years). Newfound evidence of sub-haplogroup V2 (PP = 1) has a more recent coalescence age dated back to 1.5 kya (95% HPD 690–2788 years), while sub-haplogroup V1 (PP = 0.97) diversified earlier (2.3 kya; 95% 1005–3815 years).

## Discussion

The timing and location of chicken domestication have been the subject of protracted debate worldwide and have stimulated several molecular studies using modern biological and zooarchaeological data^8,9,11–13,20,31^. The consensus among researchers and several molecular studies confirmed that domestic chickens evolved from red junglefowl somewhere in South and Southeast Asia^7,8,11,28,31^, but identifying their exact geographic center of origin has been challenging^9,12,13^. Here, we present a comprehensive resolution of mitochondrial lineage diversity and phylogenetic analyses, population differentiation, demographic inference, and divergence time estimates of chickens in Southeast Asia and the Pacific region. Patterns of sequence variation indicated that chickens in the MSEA region have higher intrapopulation genetic diversity than island populations. The average genetic diversity values of Southeast Asian chickens (MSEA: *Hd* = 0.963 ± 0.005; *π* = 0.00782 ± 0.00398; ISEA: *Hd* = 0.942 ± 0.009; *π* = 0.00466 ± 0.00249) observed in this study were higher than those of Chinese chickens (*Hd* = 0.893 ± 0.011; *π* = 0.00591 ± 0.00111)^31,38^, Japanese chickens (*π* = 0.00162 ± 0.00103)^39,40^, South Asian chickens (i.e., India: *Hd* = 0.582–0.737^28,31^; Bangladesh: *Hd* = 0.921 ± 0.018; *π* = 0.0061 ± 0.0019^41^; and Pakistan: *Hd* = 0.825 ± 0.051; *π* = 0.00536 ± 0.00075^42^), Egyptian chickens (*Hd* = 0.81 ± 0.03; *π* = 0.0045 ± 0.0013)^43^, and East African chickens (*Hd* = 0.638 ± 0.024; *π* = 0.00745 ± 0.00042)^32^. The substantial diversity of SEA chickens reflects the high matrilineal genetic variation documented in the major haplogroups, particularly haplogroup D with a large number of divergent haplotypes and haplogroup V, which has been detected only in Thailand, Cambodia, and Laos (Supplementary Tables S2, S3). However, we cannot invalidate the influence of RJFs samples on the overall genetic diversity as they reflect ancestral genetic variations. Divergent sub-haplogroups that retained ancestral variations were also observed in these lineages, likely due to geographic proximity to the center of domestication. These defined indices of biodiversity offer great opportunities for developing genetic improvement strategies, trait selection, effective management of genetic resources, and future conservation efforts^1,4,44,45^.

Pioneering molecular studies and DNA sources based on the hypervariable region (partial sequence)^7^, complete mtDNA control region^8,39^, mitogenome^8,31^, and whole-genome data^11,20,22^ provided important insights in resolving the chicken phylogeny. In addition, recent genome-wide phylogenetic inferences provided a new perspective of wild species ancestry (i.e., *G*. *g. spadiceus*) of domestic chickens in southwestern China and Southeast Asia^11^. However, topological discrepancies have also been documented in genome-wide data, often explained by differences in data sources and taxon sampling^20,22,46^. The scope of the present study defines new evidence for modern chicken genetic information with increased data sources spanning Southeast Asia and Oceania. Furthermore, zooarchaeological DNA analysis can further clarify the evolutionary history of chickens in this region^12,47^.

Population genetic and phylogenetic analyses of more than 500 complete mtDNA control region sequences unveiled new perspectives on the population dynamics of SEA and Pacific chickens. Consistent with reports from various population genetic analyses, haplogroups A and B were widely distributed in East and Southeast Asia, while haplogroup E had the widest global distribution^7,8,19,31,40^. Haplogroup F was primarily represented in Myanmar chickens and shared this matriline with chicken populations in adjacent Yunnan Province, China^7,31,34^. Consistent with the phylogenetic analyses, the pairwise *F*_*ST*_ value of Myanmar chickens was not genetically different from those of Yunnan chicken populations (Fig. 3a–b; Supplementary Table S6). This can be explained by the geographic proximity and the course of the Burma Road, which connects Myanmar and Yunnan Province^48^. Genetic differentiation of populations and PCoA analyses revealed genetic substructure between geographically isolated populations, i.e., between MSEA and ISEA chickens, South Asian and East Asian chickens, and South Asian and ISEA chickens (Fig. 3a; Supplementary Table S6). Transregional population substructure was also observed within Southeast Asian chickens, reflecting deep phylogeographic diversification. Strong topological supports consistently define major haplogroup nomenclatures and provide evidence for the presence of a haplogroup D ancestral lineage (i.e., sub-haplogroup D2) from MSEA populations. A new matrilineage (i.e., sub-haplogroup V2) gave rise to the population of domestic chickens sampled in Cambodia, Laos, and Thailand, whereas their ancestral lineage (i.e., sub-haplogroup V1) was represented in Thai red junglefowl (i.e., *G*. *g*. *gallus*). The previously reconstructed mtDNA phylogenetic tree described by Huang et al.^31^ assigned some of the previously identified haplogroup C samples to haplogroup V and linked them as a sister clade to the macrohaplogroup CD. However, because of the expanded sample distribution and increase in samples, we characterized haplogroup V as a sister group to haplogroup C only (Figs. 1, 2; Supplementary Fig. S2). This resulted in a clearer reclassification of macrohaplogroup CDV (Fig. 1b). Interestingly, the ancestral matrilines classified under sub-haplogroup D2 and haplogroup V were identified in sampling areas along the Lower Mekong subregion, for example, in Champasak and Bolikhamsai provinces in Laos, in Kampong Cham, Mondulkiri, Stung Treng, and Kratie provinces in Cambodia (Supplementary Table S1). The favorable climatic conditions and vegetation in this area are suitable for the red junglefowl (and their earlier descendants) to diversify and expand their distribution within their native range^6,10,49^. Collias & Saichuae^50^ observed that the bird is drawn to primitive agriculture and converted primary forest. The bird also thrives and populates in the bamboo forest with lower elevations as well as near water holes or streams. In addition, migratory junglefowls have been sighted in the areas closer to the Mekong River, apparently attempting to cross it: *“In crossing, the birds fly up as high as they can go, and then attempt to glide across… This movement does not seem to be caused by lack of food as the birds are extraordinary plump and in good condition. It is not easy to understand why it is taking place, as conditions on both sides of the Mekong seem the same”*^51^. As one of the most geologically dynamic regions in the world, the Indo-Burma Biodiversity Hotspot has the highest recorded bird species (> 1,200) in the entire Asia–Pacific region^14,17^. The favorable seasonal weather patterns (i.e., dry northeast monsoon) and vegetation in much of the south, central, and west of the hotspot^17^ make it a suitable habitat for chicken dispersal^10,12,18^.

Meanwhile, the presence of the sub-haplogroup D1b classified for the Philippine and Pacific chicken populations is well documented in the present study and strongly supported by bootstrap and posterior probabilities. This matriline represents genetic uniformity and shows no significant signals of population structure despite geographic isolation between the Philippines and the Pacific region^36,37^. This recently expanded lineage is unique to this region, suggesting a human-mediated scenario of its phylogeography. This may be due to the dispersal of Austronesian speakers to the Philippines (ca. 4000 cal. BP) and continued movement eastward to the Melanesian islands (ca. 3300–3150 cal. BP) and as far as Remote Oceania^52–54^. This translocation route has been reliably defined by the recovered ancient DNA from Polynesian chickens, which identified the Philippines as a homeland for the diversity of Pacific chickens^36,55^. Similarly, the phylogeographic dispersal of the sub-haplogroup D1b, which first diversified in the Philippine archipelago, likely corresponds to the initial introduction pattern of their ancestral matriline (i.e., sub-haplogroup D2) from MSEA. This translocation pattern may have been influenced by the numerous waves of human migration to the Philippines brought by the Negritos across the continental landmass of Sundaland^56–58^. The introduction of the Manobo and Sama ancestry into the southern Philippines and Palawan cannot be ruled out, as they showed high genetic relatedness to MSEA-affiliated populations^58^. The timing of migration of people of Manobo ancestry (> 12,000 years ago) and people of Sama ancestry (~ 8000–12,000 years ago) is the closest possible translocation scenario^58^, which is consistent with archaeological evidence suggesting that the domestication of chickens in Southeast Asia occurred long before 8000 BP^6^. However, there are few reports of chicken remains in Southeast Asia^24^, and prehistoric exploitation has yet to be discovered^25^. Therefore, zooarchaeological and paleoclimatic studies are essential to identify their exact geographic center of origin reliably. On the contrary, we cannot assume a unidirectional north-to-south translocation of chickens from Taiwan because Taiwan's indigenous chickens (e.g., Ju-Chi) and gamecock (Hua-Tung) share genetic similarities with East Asian chicken haplotypes and populations introduced from Japan and the Indian subcontinent^59^.

Our coalescent-based Bayesian demographic analyses detected earlier effective population size expansion in MSEA chickens, while island populations showed more recent demographic growth signatures. Although our BSP results consider relevant sampling schemes with high sample sizes per demes, we still carefully acknowledge the potential impact of population structure on demographic estimates^60^. The timing of the demographic expansion of MSEA chickens observed in this study can be explained by the cultural importance of stock-raising in the archaeological sites of Non Nok Tha and Ban Chiang in Thailand around ca. 4000–3000 BP^49^. Bones of animals (e.g., pig, cattle, dog, deer, and chicken) and clay animal figurines were excavated in the human burial sites, suggesting that animals were part of the ritual practices during prehistoric inhumation^49^. It was well documented that agriculture and animal-raising were among the subsistence activities of domestic communities during prehistoric settlements in the broad valleys of the Lower Mekong^49^. In addition, ancient DNA of Thai chickens that were recovered in Ban Non Wat dated back to around 2500 BP, also supported the demographic expansion of MSEA chickens^24^. Recent morphological bone identification further documented the existence of chicken remains from other known archaeological sites in Thailand as early as 4000 BP^25^. On the other hand, the demographic expansion pattern of the island chicken population seems to suggest the timeline of Austronesian settlement in the region^61,62^.

The time tree phylogeny in the coalescent framework allowed us to estimate nodal ages of haplogroups relevant to this study. We combined primary calibration (i.e., fossil) from ancient Pacific chickens^36^ and secondary calibration from previous estimations by Lawal et al.^20^. The latter calibration can provide close derived estimates from true time depending on the type of primary calibrations used^63,64^. Modelling the minimum–maximum constraints allows proximate measurement of uncertainties for estimated times and includes true time boundaries in the derived time CI range^63–66^. These calibrations and our robust phylogenetic trees allowed us to estimate the divergence of major haplogroups and the coalescence ages of some lineage-specific matrilines that shaped the population demographics of Southeast Asian chickens. For example, the coalescence time estimate for the node of macrohaplogroup CDV is estimated around 6.67 kya (95% HPD: 4235–7996 years). A similar age estimate was reported by Huang et al.^31^ under a relaxed molecular clock model using the same molecular rate. The evidence of earlier coalescence age of haplogroup D ancestral matriline (i.e., sub-haplogroup D2) from MSEA exemplified dispersal patterns to the ISEA, and thereafter island clade diversified as a distinct group, a phylogeographic scenario that was also documented in other avian taxa^15,67,68^. Earlier paleoenvironment and biogeographic evidence^69^ and more recent evidence on stable carbon isotope records from bat guano sequences^70^ suggest that seasonal forest or open vegetation existed in the continental landmass of Sundaland during the Last Glacial Period, which likely facilitated early human dispersal through the region^71^. The most recent common ancestor (TMRCA) of modern Philippine-Pacific chickens (i.e., sub-haplogroup D1b) and the ancient Pacific samples date back to 2.1 kya (95% HPD 1467–2815 years). This estimate predates the sample ages of the recovered ancient Pacific chickens in Anatoloa site, Niue Island and Anakena site, Rapa Nui^36^. The age estimate of haplogroup V indicates an older coalescence age (3.9 kya; 95% HPD: 2125–5880 years) than the previous estimate of this reclassified haplogroup^31^. Caution is warranted for this interpretation because the coalescence age estimate of gene copies in ancestral populations is not equivalent to a population split^72,73^, nor does it represent the actual onset of domestication.

In conclusion, this study provides a comprehensive insight into the genetic diversity and unique population dynamics of Southeast Asian chickens. High-resolution matrilineal phylogeny sheds new light on the evolutionary history of globally acknowledged haplogroups of SEA and Pacific chickens. It provides evidence of a new divergent matrilineage (i.e., haplogroup V) distributed across its native range in the Lower Mekong subregion. The phylogeographic and time tree phylogeny suggests human-mediated translocation of the haplogroup D ancestral matriline (i.e., haplogroup D2) from MSEA, which later diversified, forming a divergent sub-haplogroup D1b distinct to the island populations (i.e., Philippine-Pacific subclade). Future integrated genome-wide and environmental adaptation studies are required to unravel new elements of genomic evolution of SEA chickens for sustainable genetic improvement for climate resilience, effective management strategies, and future conservation endeavors.

## Materials and methods

### Ethics statement

Animal care and experimental procedures were approved by the Institutional Animal Care and Used Committee Guidelines of Hiroshima University as established by the Laboratory of Animal Genetics, Graduate School of Integrated Sciences for Life (Approval No. 015A170426). All blood sample collections were conducted following the fundamental Guidelines on the Use of Experimental Animals of the Laboratory of Animal Genetics, Hiroshima University, Japan.

### Sampling and DNA extraction

Blood samples were collected from a total of 369 individuals from Cambodia (*n* = 173, domestic chickens), Laos (*n* = 63, domestic chickens), Myanmar (*n* = 75, domestic chickens; *n* = 3, red junglefowls), Thailand (*n* = 18, red junglefowls; *n* = 7, domestic chickens), Philippines (*n* = 6, red junglefowls), and Fiji, Melanesia (*n* = 24, domestic chickens) (Supplementary Fig. S1). Details of the sampled animals and their geographical distribution are listed in Supplementary Table S1. Sampling was carried out from the unrelated individuals (e.g., sampling from a different known family and different sites within every province) to avoid lineage contamination during the later analyses. Genomic DNA was extracted from stored whole blood samples using the phenol–chloroform method^74^.

The final dataset was complemented with previously published sequences of Philippine chickens (*n* = 129)^37^ and directly submitted sequences of Indonesian (*n* = 10) and Pacific chickens (*n* = 11) retrieved from GenBank (Supplementary Table S1).

### PCR amplification and sequencing

The target complete mtDNA control region (1232 bp) was amplified in two procedures. First, about 5.0 kb mtDNA D-loop fragments were amplified using a long and accurate—PCR (LA-PCR) kit (KOD-FX Neo Polymerase, TOYOBO, Osaka, Japan) with chicken DNA as a template and LA-PCR primer sets: *Cytb-Forward*: 5ʹ-TACACGAATCAGGCTCAAACAACCCCCTAGGCATC-3ʹ, *16S-Reverse*: 5ʹ-TGCACCATTAGGTTGTCCTGATCCAACATCGAGGT-3ʹ recommended by Nishibori et al.^75^. The reaction began with a preliminary denaturation at 94 °C for 2 min, followed by 30 cycles of DNA denaturation at 98 °C for 10 s, annealing of primers at 57 °C for 30 s, and primer extension at 68 °C for 2 min and 30 s, using a GeneAmp PCR System 9700 (Applied Biosystems, Foster City, USA). Second, the amplified fragments were used for segmental amplification of the complete mtDNA D-loop region (1.3 kb) following the primer sets: *Gal1F* 5ʹ-AGGACTACGGCTTGAAAAGCCATTG-3ʹ and *Gal1R* 5ʹ-GCTGAGTACCCGTGGGGGTGTGGCT-3ʹ in 20 μl reaction volume containing 2 × PCR buffer, 0.4 mM dNTPs, 0.3 μM concentrations of each primer, 0.4 U of KOD-FX Neo DNA Polymerase, and 15–25 ng of amplified fragment DNA as template. The PCR cycling condition began with a preliminary denaturation at 94 °C for 2 min, followed by 30 cycles of DNA denaturation at 98 °C for 10 s, annealing of primers at 59 °C for 30 s, and primer extension at 68 °C for 30 s, using a GeneAmp PCR System 9700 (Applied Biosystems, Foster City, USA). The DNA fragments obtained from the segmental amplification were cleaned and purified using Exonuclease I (ExoI) and Shrimp Alkaline Phosphatase (SAP) to degrade the residual PCR primers and dephosphorylate the remaining dNTPs, respectively. The two PCR primers and one internal primer: *Gal1-2F* 5ʹ -TCCACCTCACGAGAGATCAGCAACCC-3ʹ^76^ were used for the sequencing reaction. Subsequently, the mtDNA D-loop fragments were directly sequenced using 3130/3130xl Genetic Analyzers (Applied Biosystems, Foster City, USA).

### Sequence alignment

Three hundred sixty-nine complete mtDNA control region sequences generated in this study were initially edited using GeneStudio Pro tool (GeneStudio, Inc., http://genestudio.com/). Ambiguous sites were trimmed, and cleaned sequences were aligned in MEGAX^77^ with ClustalW^78^. Aligned nucleotide sequences were viewed using BioEdit 7.2.5 software^79^. All newly generated sequences were deposited in the GenBank database with accession numbers OM240181-OM240549 (Supplementary Table S1).

### Genetic diversity and phylogenetic inference

Intrapopulation level and intraclade genetic diversity indices such as the number of haplotypes (Ht), haplotype diversity (*Hd*), and nucleotide diversity (*π*) were estimated using the DnaSP v6.0 software^80^.

Phylogenetic analyses were inferred using two different model-based approaches: maximum-likelihood (ML) and Bayesian inference (BI). Maximum-likelihood analysis was performed in IQ-TREE^81^ with the best-fit substitution model, TIM2 + F + I + G4, based on the Bayesian Information Criterion (BIC) determined by Modelfinder^82^. Statistical node support was calculated using ultrafast bootstrap support^83^ and SH-aLRT^84^ with 1,000 replicates. Bayesian inference was performed using BEAST2 v2.6.6^85^ under uncorrelated relaxed clock log-normal distribution setting a clock rate 3.13 $\times 10^{ - 7}$ mutations/site/year rate^86^. We used a general time reversible (GTR) nucleotide substitution site model with assumed rate heterogeneity among sites modeled under gamma distribution and a coalescent-based model as a tree prior. The second-best model in BIC (GTR model) was implemented because the TIM2 model is not available in BEAST2 v2.6.6. We estimated posterior distributions of parameters via Markov chain Monte Carlo (MCMC) with duplicate runs of 50 million generations, sampling every 10,000 steps, and the initial 10% trees of each MCMC run were discarded as burn-in. Convergence of MCMC chains was assessed using Tracer v.1.7.1 and sufficient sampling was verified with all estimated parameters exceeding 200 ESS values. A maximum clade credibility (MCC) tree (target tree) was obtained from a sample of trees using TreeAnnotator v2.6.3^85^. Phylogenetic trees were visualized and edited in FigTree v1.4.4. (http://tree.bio.ed.ac.uk/software/figtree/).

Median-joining network was constructed to infer the evolutionary relationships between haplotypes using PopArt v1.7 software^87^. The number and assignment of haplotypes were determined using DnaSP v6.0 software. The definition of haplogroups was employed in DomeTree (http://dometree.kiz.ac.cn/) and MitoToolPy (http://mitotool.kiz.ac.cn/)^88^.

### Population genetic structure and demographic inference

The population pairwise net genetic distance based on population pairwise *F*_ST_ (significant values were accepted at *p* < 0.05) was estimated using Arlequin v3.5.2.2 software (with 10,000 permutations)^89^. Population pairwise *F*_ST_ values were plotted into the principal coordinate analysis (PCoA) using GenAlEx v6.503^90^ to visualize the pattern of genetic relationships between geographical populations. Estimation of the genetic structures was calculated by the analysis of molecular variance (AMOVA) as implemented by Arlequin v3.5.2.2 software. The level of significance was evaluated based on 1,000 random permutations.

Inference for the population growth model was initially estimated by statistical neutrality tests, such as Tajima’s *D*^91^ and Fu’s *Fs* statistics^92^. These population expansion tests measure haplotype frequencies under neutrality. Statistical tests and confidence intervals were based on a coalescent simulation algorithm under a neutral infinite-site model. To further support the inference for the population expansion signal, a coalescent-based Bayesian Skyline Plot (BSP) was cautiously used to quantify the relationship between genealogies and the demographic history of the population^93^. BSP was simulated to infer deeper insights into the demographic history of Southeast Asian and Pacific chicken populations as implemented in BEAST v2.6.3^85^. BSP was generated with a relaxed molecular clock model and setting with 3.13 $\times 10^{ - 7}$ mutations/site/year rate^86^. The piecewise constant function and HKY + G4 nucleotide substitution model as determined by BIC in jModelTest v2.1.1^94^ was used for the analysis. The MCMC chain was run for 5 $\times {10}^{7}$ generations, with a sampling of parameters every 5000 steps and 5 $\times {10}^{6}$ generations served as burn-in. Convergence of the posterior estimates of the effective population size (*N*_*e*_) to the likelihood stationary distribution was evaluated using Tracer v1.7.1 software^95^.

### Divergence time estimate

Bayesian analyses were performed to estimate divergence times using the program BEAST2 v2.6.6. We employed a relaxed molecular clock model, which allows substitution rates to vary across branches setting with 3.13 $\times \;10^{ - 7}$ mutations/site/year rate^86^ under uncorrelated lognormal distribution and GTR + G4 substitution model as determined by BIC in jModelTest v2.1.1. We set a coalescent-based constant population to model the tree prior. The ancient DNA records of Polynesian chickens were used to calibrate the crown node of sub-haplogroup D1b (Philippine-Pacific sub-clade) (Supplementary Table S1). For this calibration point, we used a lognormal prior (mean: 2.5, SD: 0.20, offset: 0) with the maximum age of the archaeological record set as the minimum bound for the crown calibration^63,65,66,96^. For the calibration of the root node of the tree, we used the established divergence time between red junglefowl and domestic chickens (8093 years CI: 7014–8768)^20^ as a secondary calibration. We used a lognormal prior (mean: 8.09, SD: 0.05, offset: 0) covering the confidence interval range of the divergence time estimate^63,66^. Time tree analysis was run for 50 million generations, sampling every 5000 generations, and the initial 10% trees of each MCMC run were discarded as burn-in. The resulting log files were examined in Tracer v1.7.1 software^95^ to confirm acceptable mixing and convergence of all parameters in the independent runs and adequate effective sample sizes (ESS > 200). The MCC tree was created from the tree file using TreeAnnotator v2.6.3^85^ with the posterior probability set to 0.5 and common ancestor node heights summarized. These results were visualized as a single tree in FigTree v1.4.4. (http://tree.bio.ed.ac.uk/software/figtree/).

## Supplementary Information

Supplementary Information 1.

Supplementary Information 2.

## Data Availability

The complete mtDNA D-loop sequences are deposited and available in GenBank database (Accession Numbers: OM240181–OM240549).
