# Supplementary material for: Unveiling new perspective of phylogeography, genetic diversity, and population dynamics of Southeast Asian and Pacific chickens
Source: Sci Rep. 2022 Aug 26;12:14609. doi: 10.1038/s41598-022-18904-3 (PMC9418149; doi:10.1038/s41598-022-18904-3)

Unveiling new perspective of phylogeography, genetic diversity, and population dynamics of Southeast Asian and Pacific chickens

Cyrill John P. Godinez[1,2*], John King N. Layos[1,3], Yoshio Yamamoto[1], Tetsuo Kunieda[4], Monchai Duangjinda[5] Lawrence M. Liao[6], Xun-He Huang[7] & Masahide Nishibori[1,2*]

[1]Laboratory of Animal Genetics, Graduate School of Integrated Sciences for Life, Hiroshima University, Higashi-Hiroshima 739-8528, Japan

[2]Department of Animal Science, College of Agriculture and Food Science, Visayas State University, Visca, Baybay City, Leyte 6521, Philippines

[3]College of Agriculture and Forestry, Capiz State University, Burias, Mambusao, Capiz 5807, Philippines

[4]Faculty of Veterinary Medicine, Okayama University of Science, Imabari, Ehime 794-8555, Japan

[5]Department of Animal Science, Faculty of Agriculture, Khon Kaen University, Khon Kaen 40002, Thailand

[6]Laboratory of Aquatic Botany, Graduate School of Integrated Sciences for Life, Hiroshima University, Higashi-Hiroshima 739-8528, Japan

[7]School of Life Sciences, Jiaying University, Meizhou 514015, China

*** Correspondence:**
Cyrill John P. Godinez
cyrilljohn.godinez@vsu.edu.ph
https://orcid.org/0000-0002-4890-3271

Masahide Nishibori
nishibo@hiroshima-u.ac.jp
https://orcid.org/0000-0002-8378-4490

Supplementary Figure S1. Sampling locations and distribution of SEA and Pacific chickens used in this study. Red labelled complementary samples were retrieved from GenBank. The base map was created using ArcGIS 10.4.1 (https://www.esri.com/).

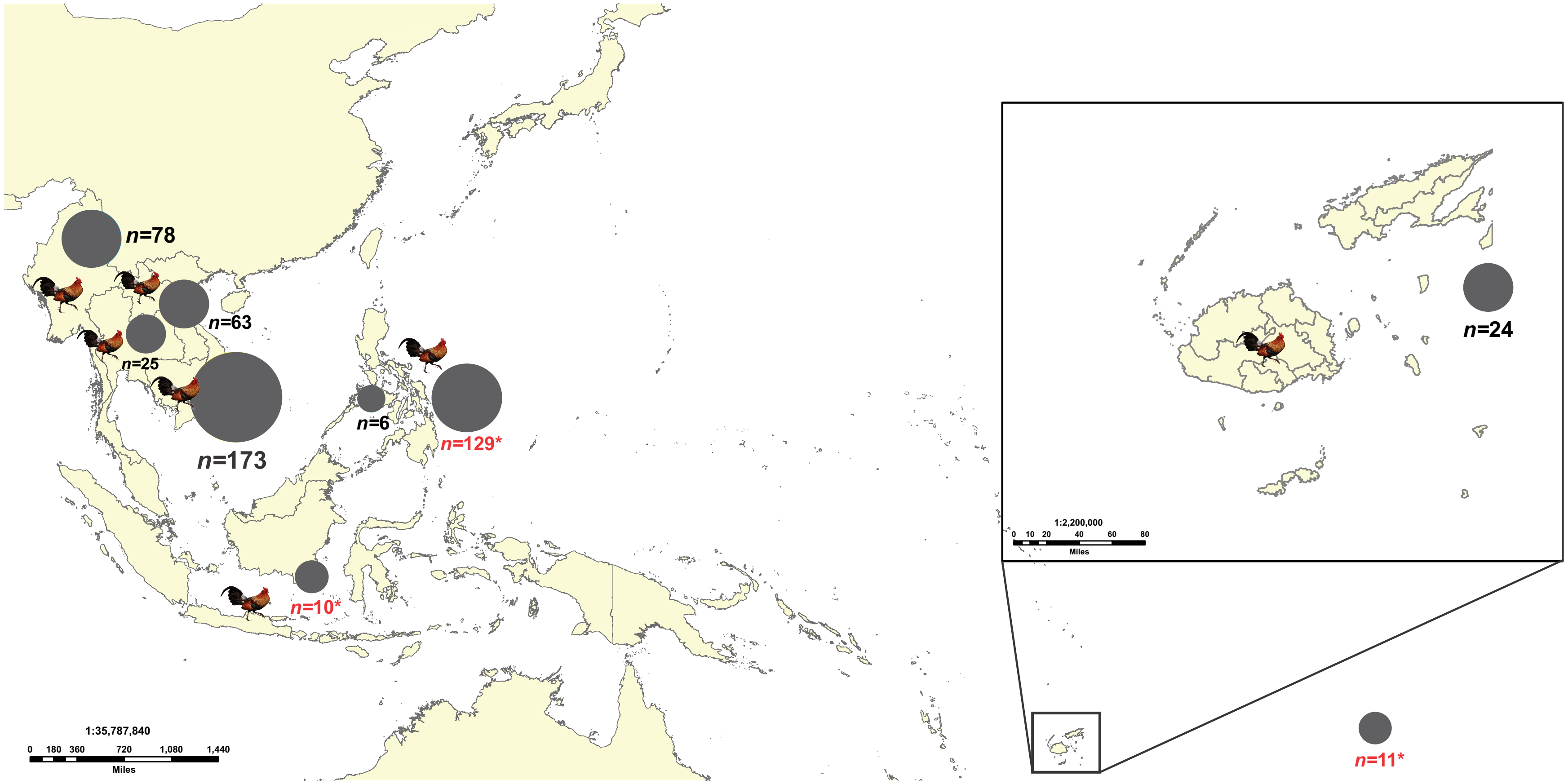

Supplementary Figure S2. Maximum likelihood phylogenetic tree for complete mtDNA D-loop nucleotide sequences of SEA and Pacific chickens. Node labels correspond to SH-aLRT/UFBoot support values evaluated with 1,000 ultrafast bootstrap replicates in IQ-TREE. The scale bar (0.04) indicates the genetic distance (substitution per site). Bootstrap values under 50% are not shown. Tree file was visualized and edited in FigTree v1.4.4. (http://tree.bio.ed.ac.uk/software/figtree/).

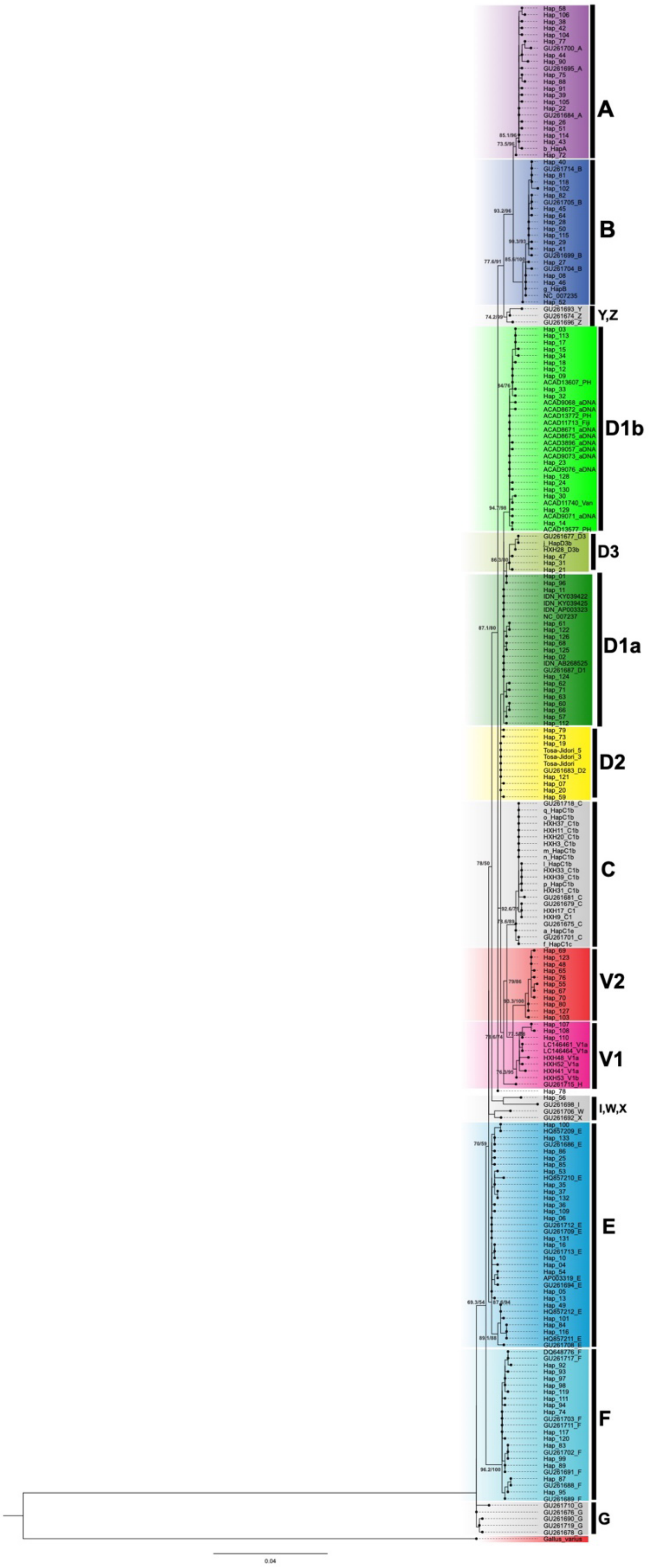

Supplementary Figure S3. Bayesian phylogenetic tree of complete mtDNA D-loop nucleotide sequences of Southeast Asian and Pacific chickens showing position of terminals. The tree was constructed together with database sequences defined by Huang et al. (2018) (Supplementary Table S4). Node labels correspond to posterior probability support values. *Gallus varius* was used as outgroup. Tree file was visualized and edited in FigTree v1.4.4. (http://tree.bio.ed.ac.uk/software/figtree/).

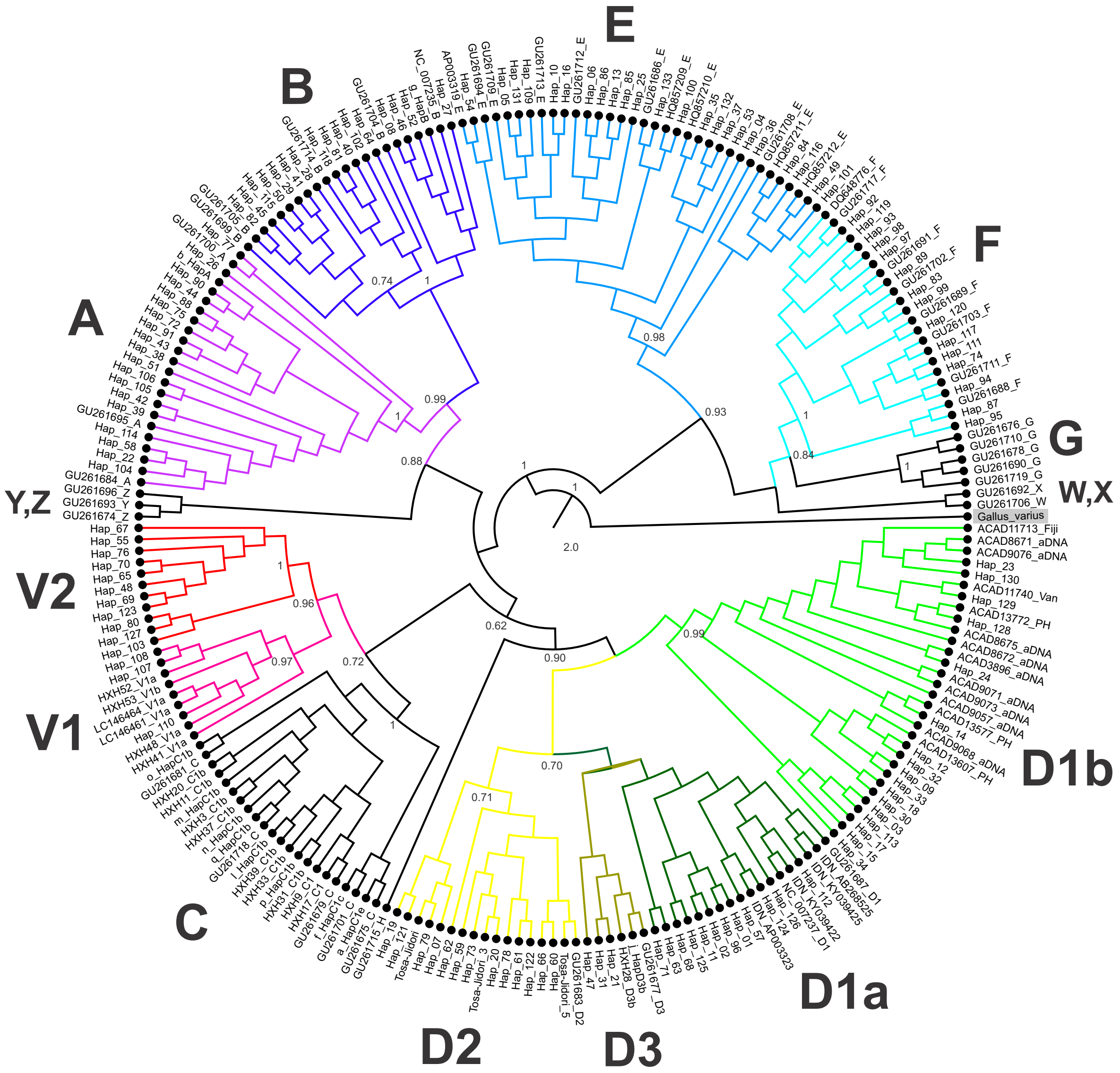

Supplementary Figure S4. Geographic-specific median-joining network of the complete mtDNA D-loop region (1,232 bp) depicting within population evolutionary relationship. The area of each circle is proportional to the frequency of the corresponding haplotypes. The length of branch connecting to other haplotypes corresponds to mutational positions. The base maps were created using ArcGIS 10.4.1 (https://www.esri.com/).

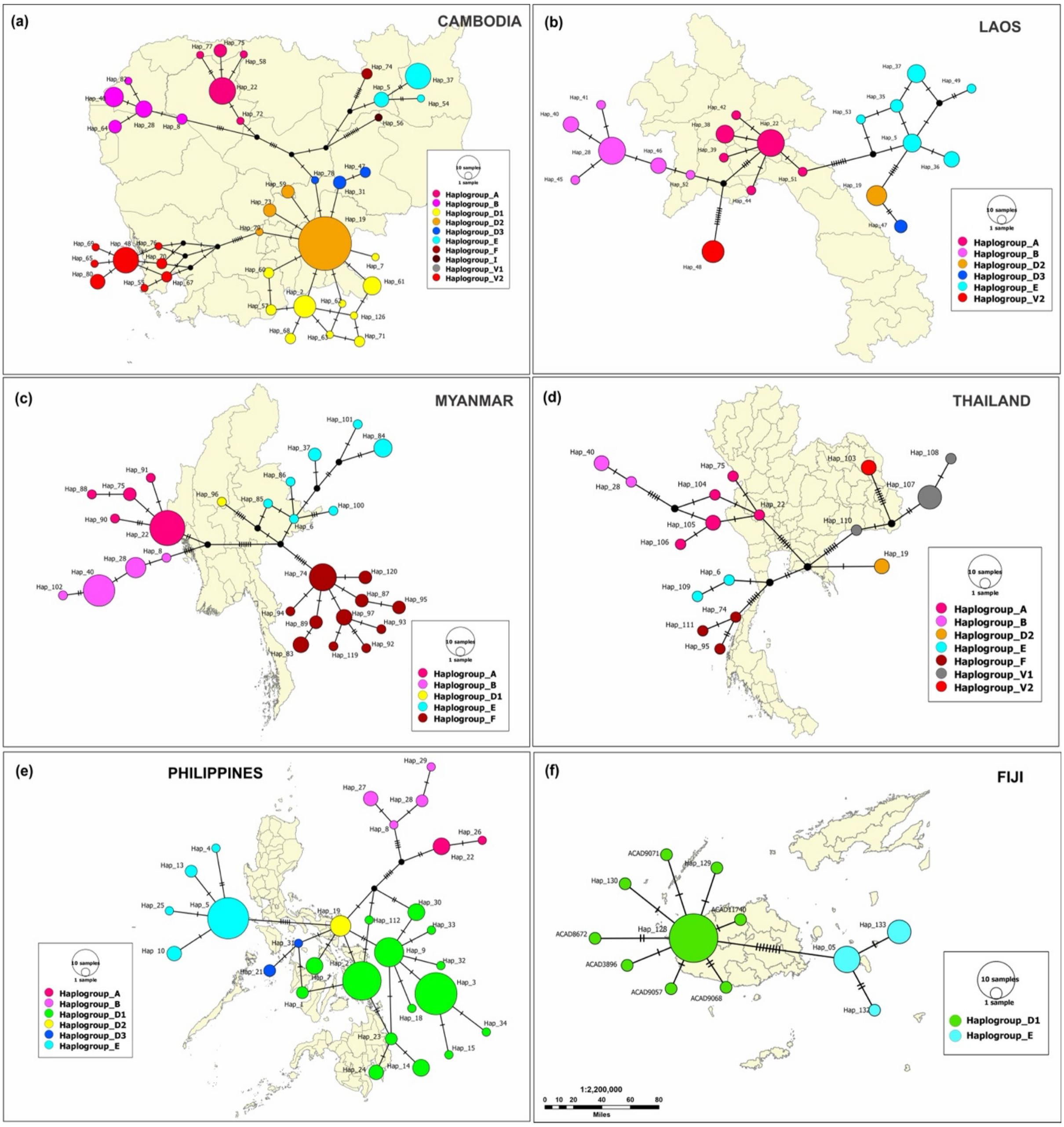

Supplementary Figure S5. Geographic-specific Bayesian coalescent skyline plot showing estimated within population demographic history. The central blue line is the median estimate effective population size. The shaded area shows the upper and lower estimates of 95% credibility interval. The x-axis is the time (in years before present) and y-axis indicates population size (as the product of N_e and the generation length in years).

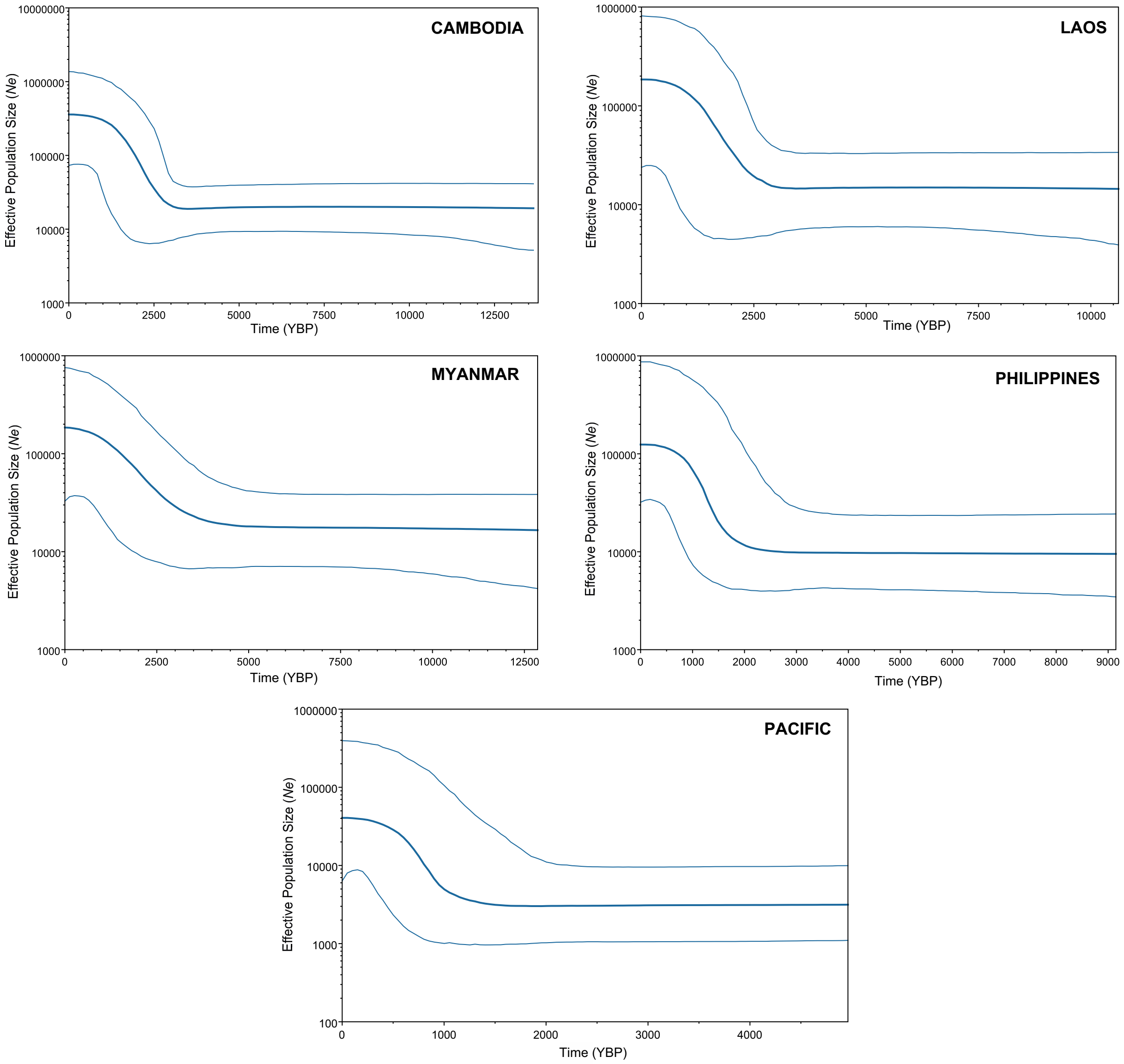

Supplement: Supplementary file 1 — Supplementary Information 1. [file 41598_2022_18904_MOESM1_ESM.pdf]
